# Antibody Response to *Mycoplasma pneumoniae*: Protection of Host and Influence on Outbreaks?

**DOI:** 10.3389/fmicb.2016.00039

**Published:** 2016-01-26

**Authors:** Roger Dumke, Enno Jacobs

**Affiliations:** Institute of Medical Microbiology and Hygiene, Technische Universitaet DresdenDresden, Germany

**Keywords:** *Mycoplasma pneumoniae*, community-acquired pneumonia, immune response, adherence, protective antibodies

## Abstract

In humans of all ages, the cell wall-less and genome-reduced species *Mycoplasma pneumoniae* can cause infections of the upper and lower respiratory tract. The well-documented occurrence of major peaks in the incidence of community-acquired pneumonia cases reported world-wide, the multifaceted clinical manifestations of infection and the increasing number of resistant strains provide reasons for ongoing interest in the pathogenesis of mycoplasmal disease. The results of recent studies have provided insights into the interaction of the limited virulence factors of the bacterium with its host. In addition, the availability of complete *M. pneumoniae* genomes from patient isolates and the development of proteomic methods for investigation of mycoplasmas have not only allowed characterization of sequence divergences between strains but have also shown the importance of proteins and protein parts for induction of the immune reaction after infection. This review focuses on selected aspects of the humoral host immune response as a factor that might influence the clinical course of infections, subsequent protection in cases of re-infections and changes of epidemiological pattern of infections. The characterization of antibodies directed to defined antigens and approaches to promote their induction in the respiratory mucosa are also preconditions for the development of a vaccine to protect risk populations from severe disease due to *M. pneumoniae*.

## Immunodominant Antigens of *M. pneumoniae* and Host Response

In addition to the role of virulence factors of *M. pneumoniae*, the induction of specific antibodies is not only important for serodiagnosis of infections but also for the colonization process. As result of lacking the classical bacterial cell wall, components of the cell membrane are important for interaction of the pathogen with the host. The importance of an intact immune response is demonstrated by the increased occurrence of severe disease, repeated infections or prolonged persistence of *M. pneumoniae* in patients with deficiencies of humoral immunity (Foy et al., 1973; Taylor-Robinson et al., 1980; Roifman et al., 1986), thus emphasizing the role of specific antibodies for protection. The antigens of *M. pneumoniae* cells determining the host response include glycolipids as well as proteins (Morrison-Plummer et al., 1986) that induce comparable immune reactions in affected individuals (Jacobs et al., 1986; Vu et al., 1987). In comparison with glycolipids, the more specific proteins were mainly characterized as components of the adhesion apparatus of *M. pneumoniae* (Razin and Jacobs, 1992). In particular, antibodies to the P1 protein are regularly found in sera of infected patients. The large membrane protein (168 kDa) was characterized as the main adhesin of the bacteria and is also the most antigenic protein, inducing strong and early production of antibodies (Hu et al., 1983). Using different proteomic approaches such as fractionation of whole proteins (Regula et al., 2001), construction of a whole-genome phage display library (Beghetto et al., 2009) or 2D separation of proteins followed by incubation with sera of infected patients (Nuyttens et al., 2010) resulted in the characterizations of further antigens which are membrane-associated and potentially interact with the host immune system. Besides proteins with a confirmed function in adherence, putative lipoproteins, glycolytic enzymes (e.g., pyruvate dehydrogenase subunit B), chaperones (GroEL, DnaK) and proteins of translation/transcription (e.g., elongation factor Tu) were found. Some of these proteins are surface-localized and involved in interactions with components of the human extracellular matrix (Dallo et al., 2002; Gründel et al., 2015). In addition, CARDS toxin as an important virulence factor of *M. pneumoniae* was characterized as an immune-dominant protein (Kannan and Baseman, 2006). However, the role of antibodies to many of these proteins for the potential to protect the host from re-infections remains to be proved.

With the development of specific tools for investigation of mycoplasmas (Halbedel and Stülke, 2007), such as targeted mutation of TGA triplets coding for tryptophan in *M. pneumoniae* (Inamine et al., 1990), the recombinant production and analysis of proteins of interest for host–pathogen interaction have accelerated. Regarding naturally infected hosts, **Table 1** summarizes defined *M. pneumoniae* proteins that were found in recent years to elicit a specific and strong immune reaction in humans. These studies confirmed that the immune response is dominated by antibodies against the adhesins and adhesion-related proteins of the bacterium that have limited effect on viability (Krause and Baseman, 1983). It can be suggested that the antibody response results mainly in an influence on the gliding process (Seto et al., 2005) and a decrease of adhesion of bacteria to the target cells of the respiratory mucosa. Studies using quantitative methods to measure the adherence of *M. pneumoniae* to human cells *in vitro* showed that specific antisera to total proteins, to adhesins or even to defined regions of adhesins are able to inhibit adhesion to more than 90% in comparison with control sera (Svenstrup et al., 2002; Schurwanz et al., 2009). The importance of the adherence process for further colonization is underlined by the fact that mutants defective in expression of different adhesins and adhesion-related proteins are avirulent (Balish and Krause, 2006).

**Table 1:** Recombinant *Mycoplasma pneumoniae* proteins tested as antigens for detection of specific antibodies in humans.

Protein (gene)	Function	Remarks	Reference
P1 (*mpn141*^∗^)	Adherence	Use of C-terminal protein part	Drasbek et al., 2004
		Full-length characterization using 15 recombinant proteins, construction of a chimeric protein of C-terminal P1 part and P30	Schurwanz et al., 2009
		Construction of a chimeric protein of C-terminal P1 region and parts of P30 and MPN456 (unknown function)	Montagnani et al., 2010
		C-terminal protein part	Nuyttens et al., 2010
		Conserved C-terminal and variable part of repMP4 of P1-types 1 and 2	Dumke et al., 2012
		C-terminal protein part	Xue et al., 2013
		Immunodominant COOH epitope	Wood et al., 2013
		Full-length characterization by using four recombinant proteins	Chourasia et al., 2014
P30 (*mpn453*)	Adherence	Full-length protein	Varshney et al., 2008
		Fragment of P30 (without N-terminus)	Schurwanz et al., 2009
P90 (*mpn142*)	Adherence	Fragment of P90 (aa 751–1088)	Dumke et al., 2012
P200 (*mpn567*)	Adherence	Fragment of P200 (aa 641–678)	Dumke et al., 2012
AtpD (*mpn598*)	Energy metabolism	Full-length protein	Nuyttens et al., 2010
CARDS toxin (*mpn372*)	Cytotoxin	Full-length protein	Wood et al., 2013
P116 (*mpn213*)	Hypothetical (adherence?)	Protein fragment (53 kDa)	Duffy et al., 1999
		Protein fragment (without C-terminus)	Drasbek et al., 2004
		N-terminal protein region (27 kDa)	Tabassum et al., 2010
P400 (*mpn400*)	Hypothetical	Fragment of P400 (aa 407–582)	Dumke et al., 2012


*^∗^According to Dandekar et al., 2000.*

Beside problems in the sensitivity and specificity of serological assays (Loens et al., 2010; Busson et al., 2013), *M. pneumoniae* infections are complicated by different host-dependent characteristics, such as variable persistence of antibodies, missing IgM response after re-infection and the infrequent production of IgA antibodies in children (Atkinson et al., 2008). IgM antibodies can be detected 7–10 days after infection and IgG immunoglobulins are measurable approximately 14 days later (Atkinson et al., 2008; Atkinson and Waites, 2014).

## Genotype-Specific Immune Response and Influence on the Epidemiology of *M. pneumoniae* Infections

Genome plasticity and different mechanisms for antigen variation are a typical pattern of different mycoplasma species with pathogenic potential (Citti and Blanchard, 2013). In *M. pneumoniae*, recent studies comparing whole genomes resulted in a remarkable homology between strains of different origin and isolation period (Lluch-Senar et al., 2015; Xiao et al., 2015). However, isolates of *M. pneumoniae* or strains in respiratory tract samples from patients show defined sequence variations which can be used for typing by different methods. Multilocus variable number of tandem repeat analysis (Degrange et al., 2009), multilocus sequence typing (Brown et al., 2015) and SNP minisequencing (Touati et al., 2015) have been developed recently using current molecular tools. These approaches investigate variable regions in the genome which are located mainly in intergenic regions and in genes coding for hypothetical proteins or for house-keeping proteins with an intracellular function. Furthermore, the latter two approaches detect small polymorphic sites and even single nucleotide exchanges. Regarding the immune response, an influence of these differences on protective antibodies in infected hosts can hardly be expected. Also, the role in the immune reaction of more different genome regions such as certain genes that contain copies of the repetitive element RepMP1 (Musatovova et al., 2012) is still unclear as the gene products involved and their possible functions have yet to be investigated. Lipoproteins are important antigens in many mycoplasma species showing variations to escape the host immune response (Citti et al., 2010; Szczepanek and Silbart, 2014). In *M. pneumoniae*, a high proportion (nearly 7%) of genes encoding for putative lipoproteins was confirmed and their expression pattern was investigated (Hallamaa et al., 2006, 2008). So far, strain-specific induction of anti-lipoprotein antibodies during infection has not been demonstrated.

The genome region with the greatest discrimination power in combination with well-characterized gene products is the p1 operon coding for P1 adhesin and the adhesion-related proteins P40 and P90, as well as a phosphoesterase (*mpn140*). Copies of repetitive elements RepMP2/3 and RepMP4 (p1 gene, *mpn141*) as well as of RepMP5 (*mpn142*) can be found, which differ between isolates (Su et al., 1990). Analysis of repetitive elements distributed in variable size and sequence over the genome of *M. pneumoniae* strains resulted in the characterization of p1 type 1 and p1 type 2 (Spuesens et al., 2009). Despite a high number of RepMP2/3 (*n* = 10), RepMP4 (8) and RepMP5 (8) copies in the genome of strain M129 (Dandekar et al., 2000), the number of circulating p1 types is relatively low. It can be suggested that the complex functions of the P1 protein limit the possible recombination events that allow effective adhesion, gliding and/or division processes of mycoplasma cells. In addition to the main types 1 and 2, variants differing to a smaller extent from the two p1 types (mainly in the RepMP2/3 copy) were identified after investigation of sequence of p1 genes of clinical strains collected world-wide. According to recent knowledge, these variants cannot be distinguished in the sequence of *mpn142*, indicating lower variability of proteins P40 and P90. Despite the fact that 76% of the p1 sequence can be assigned to both RepMP copies, the divergence between the amino acid sequences of P1 adhesins of types 1 and 2 strains (1,628 and 1,635 aa) is only about 5%. However, these distinct differences in amino acid sequence may influence the immune response of a colonized host. Indeed, it was shown that the variable parts of the P1 adhesins are antigenic resulting in a type-specific response in immunized animals (Dumke et al., 2008). Thus, recombinantly produced protein parts derived from variable regions of the P1 adhesin can be used for determination of type-specific IgG immunoglobulins (Dumke et al., 2012). Furthermore, guinea pigs infected intranasally with a type 2 strain developed protective immunity to type 2 strains after re-infection with a mixture of types 1 and 2 (Dumke et al., 2004). As a consequence of these findings and because of the dominant role of antibodies to the P1 protein in sera of infected patients, it has been suggested that type-specific immunoglobulins have an influence on subsequent re-infections in affected individuals as well as on the epidemiology of infections in greater populations. The results of different studies confirmed that the epidemic peaks of respiratory infections due to *M. pneumoniae* occurring at intervals of 3–7 years were correlated with a change of the predominant p1 type (Kenri et al., 2008; Kogoj et al., 2015; Suzuki et al., 2015; Zhao et al., 2015). In other investigated populations, significant differences in the proportion of types 1 and 2 strains during endemic and epidemic periods of infections were not detected (Dumke et al., 2015; Jacobs et al., 2015). Therefore, further long-term epidemiological studies are needed to clarify whether type-specific antibodies induced after an outbreak will protect patients from a re-infection with this genotype of *M. pneumoniae*. In a study investigating type-specific antibodies in acute-phase sera (IgA and IgG) from pneumonia patients with known p1 type in the respiratory tract, correlation between the occurrence of genotypes and type-specific immune response was lacking (Dumke et al., 2010). Finally, it cannot be excluded that genotype-specific differences in adhesion-related proteins will influence the interaction with target structures for adherence. Interestingly, recent epidemiological reports describe the replacement of p1 type 2 strains by variants of this type in the investigated human population (Jacobs et al., 2015; Suzuki et al., 2015). The reasons for the shift of genotypes remain unclear since it seems unlikely that the small sequence variations in the p1 gene of type 2 and type 2 variant strains will influence the immune response of infected patients. Confirmed type-specific differences in factors that influence the interaction with the host such as biofilm formation (Simmons et al., 2013) or expression of CARDS toxin (Techasaensiri et al., 2010; Lluch-Senar et al., 2015) were demonstrated between types 1 and 2 strains but not between type 2 and type 2 variants. Further studies should be performed to give more insight into the role of the time-variable occurrence of genotype-specific antibodies (host-dependent) and the consequences of sequence differences in the P1 protein for the adherence process (pathogen-dependent) in the epidemiology of infections.

## Vaccine Development

Despite the benign course of many infections by *M. pneumoniae*, the occurrence of severe manifestations and the existence of risk populations with intensive person-to-person contacts (schools, military camps) or with chronic respiratory disease justify efforts to prevent infections. Recently, a further reason resulted from high rates of macrolide-resistant strains circulating mainly in Asia, limiting the antibiotic treatment options especially in pediatric patients. The availability of safe vaccines seems nowadays the most effective measure for control of *M. pneumoniae* also, and this is supported by the increased knowledge about the virulence factors and by the confirmed genetic homogeneity of this pathogen. Furthermore, the described induction of a strong host immune reaction to defined pathogen factors has encouraged vaccination experiments. However, the results of early studies with volunteers treated with inactivated whole antigen preparations reflect the problems of inducing an immune response that demonstrates effective protection from subsequent infections (summarized in Linchevski et al., 2009). Limited efficacy against pneumonia, adverse reactions and, in some cases, exacerbation of symptoms after infection of immunized individuals have been described and confirmed by recent animal experiments (Sekine et al., 2009; Szczepanek et al., 2012).

Based on the importance of adherence of *M. pneumoniae* cells for initiating host colonization, recent studies have focused on the induction of adhesion-blocking antibodies. Investigation of components of the adhesion complex resulted in defined protein regions which are antigenic and involved in adherence and which have a conserved sequence (Schurwanz et al., 2009; Nakane et al., 2011). The construction of chimeric antigens composed of shorter protein regions with characterized function in adherence provides an opportunity to target different adhesion-related structures (Schurwanz et al., 2009). Immunization of guinea pigs with a hybrid protein consisting of adherence-related parts of the proteins P1 and P30 led to a significant decrease of specific genome copies in respiratory tract samples from vaccinated and subsequently infected animals (Hausner et al., 2013). Since demonstration of the adherence-blocking properties of sera from immunized animals, the induction of potent stimulation of mucosal immunity can be suggested as a crucial aspect for successful vaccination. This included not only (intranasal) administration of the antigen but also combination with biocompatible adjuvants (Zhu et al., 2012; Hausner et al., 2013).

## Carriage of *M. pneumoniae*

As with other respiratory pathogens such as pneumococci (Donkor, 2013), asymptomatic or convalescent individuals have been confirmed as carriers of *M. pneumoniae* cells in the upper respiratory tract (Foy, 1993). This is an important fact, not only for the significance of laboratory test results and for epidemiological aspects of transmission but also for evaluation of the role of protective antibodies. Unfortunately, the results of carriage studies are inconsistent. Gnarpe et al. (1992) showed that culturable mycoplasmas occurred in the throats of a relatively high proportion (13.5 and 4.6%) of subjectively healthy adults in two investigation periods. In a recent study, detection of *M. pneumoniae* infections using ELISA (IgM and IgG) and real-time PCR was similar in groups of asymptomatic children and pediatric patients with respiratory symptoms (Spuesens et al., 2013). In contrast, Jain et al. (2015) reported a low rate (<3%) of PCR-positive results in asymptomatic children in a control group during a period of high incidence of *M. pneumoniae*. This is in accordance with other studies that demonstrated no detection or only low rates of respiratory tract colonization/infection of asymptomatic patients of different ages (Kumar et al., 2008; Nilsson et al., 2008; Chalker et al., 2011). Not only the occurrence of asymptomatic carriers but also the long-term persistence of *M. pneumoniae* in the respiratory tract of immunocompetent patients raises doubts regarding the efficiency of the host immune response for clearance of bacteria. Spuesens et al. (2013) reported that 21% of PCR-positive children (with or without symptoms) showed carriage of bacteria for 1–3 months. In a study of 53 patients, the mean carriage time for *M. pneumoniae* DNA was 7 weeks after disease onset (up to 7 months) after adequate therapy and confirmation of specific antibodies in corresponding sera (Nilsson et al., 2008). Despite the strong immune response after *M. pneumoniae* infection, the efficiency of specific antibodies to eliminate the bacteria from the upper respiratory tract seems limited in particular individuals. The time-dependent decrease of specific antibodies in combination with the variable, often low production of IgA immunoglobulins in the respiratory tract may contribute to re-infection and long-term carriage.

It should be noted that confirmed persistence and long-term host colonization are typical for bacteria occurring intracellularly. The hypothesis is supported by the aforementioned absence of many metabolic pathways in *M. pneumoniae*, which requires close association with the host cells for growth and reproduction. However, the intracellular localization of *M. pneumoniae* has been described in cell culture assays (Meseguer et al., 2003) but so far not *in vivo*, so the persistence of mycoplasmas in host cells as an aspect of pathogenesis remains hypothetical.

## Conclusion

The infection of immunocompetent patients with *M. pneumoniae* induces a strong antibody response which is mainly directed to surface-located proteins of the adhesion complex of the mycoplasmas. Thus, an influence of specific immunoglobulins that inhibit the adherence of mycoplasmal cells to the respiratory epithelium of the host can be expected. However, the occurrence of long-term carriage of *M. pneumoniae*, asymptomatic carriers, re-infections as well as a broad spectrum of extra-pulmonary manifestations show that the protective effect of specific serum antibodies is limited as regards the upper respiratory tract. The directed movement of bacteria to the base of the ciliated epithelium of the respiratory mucosa and the specialized adherence to target cells of the host allow the bacteria to reach a protected niche. The role of the local immune response by inhibiting adherence and the immunomodulatory effects of local inflammation on the clinical course have yet to be explained. These factors are also crucial for the development of an effective vaccine. Finally, the confirmed induction of genotype-specific antibodies raises the question of whether this has an influence on re-infections due to different genotypes of *M. pneumoniae*, which might be important for understanding the special epidemiology of a common human respiratory tract pathogen.

## Author Contributions

RD drafted the manuscript. EJ revised and approved the final manuscript.

## Conflict of Interest Statement

The authors declare that the research was conducted in the absence of any commercial or financial relationships that could be construed as a potential conflict of interest.
